# Oxytocin Suppresses Inflammatory Responses Induced by Lipopolysaccharide through Inhibition of the eIF-2α–ATF4 Pathway in Mouse Microglia

**DOI:** 10.3390/cells8060527

**Published:** 2019-05-31

**Authors:** Takayuki Inoue, Hajime Yamakage, Masashi Tanaka, Toru Kusakabe, Akira Shimatsu, Noriko Satoh-Asahara

**Affiliations:** 1 Department of Endocrinology, Metabolism, and Hypertension Research, Clinical Research Institute, National Hospital Organization Kyoto Medical Center, Kyoto 612-8555, Japan; taka2015.www@gmail.com (T.I.); yamakage@satista.jp (H.Y.); kusakabe@kuhp.kyoto-u.ac.jp (T.K.); nsatoh@kuhp.kyoto-u.ac.jp (N.S.-A.); 2 Department of Physical Therapy, Health Science University, Yamanashi 401-0380, Japan; 3 Clinical Research Institute, National Hospital Organization Kyoto Medical Center, Kyoto 612-8555, Japan; ashimats@kyotolan.hosp.go.jp

**Keywords:** endoplasmic reticulum stress, inflammation, inflammasome, microglia, oxytocin

## Abstract

Microglia maintain brain homeostasis and modulate neuroinflammation and are implicated in the pathogenesis of various neurological diseases such as Alzheimer’s disease. In this study, we found that in lipopolysaccharide (LPS)-stimulated microglia, the endoplasmic reticulum (ER) stress-related eIF-2α–ATF4 pathway plays significant roles in TNF-α and IL-6 production, as well as in the inflammasome-mediated production of IL-1β. Furthermore, our analysis revealed that oxytocin (OT), a nonapeptide synthesized in the hypothalamus, suppressed the production of these proinflammatory cytokines by inhibiting activation of the eIF-2α–ATF4 pathway. Our findings therefore suggest a novel anti-inflammatory axis of OT in activated microglia, which would be helpful for developing the novel effective strategies for regulating microglia-associated neuroinflammation.

## 1. Introduction

Microglia are the primary cell type responsible for maintaining brain homeostasis and also modulate the neuroinflammatory state [1,2]. A phenotypic shift in microglia from homeostasis to a disease phenotype induces neuroinflammation, which is closely implicated in the pathogenesis of various neurological disorders such as Alzheimer’s disease [2,3]. It is therefore critical to develop effective strategies for regulating microglial phenotypes.

Recent accumulating evidence suggests that chronic metabolic stresses, including lipotoxicity and glucotoxicity, induce a stress reaction of the endoplasmic reticulum (ER) that causes inflammation, cell death, and multi-organ dysfunction [4,5]. The stressed ER elicits an unfolded protein response (UPR) to maintain homeostasis through activation of at least three ER stress sensors: inositol-requiring enzyme 1α (IRE1α), protein kinase RNA-like ER Kinase (PERK), and activating transcription factor 6 (ATF6) [4,5]. These three branches of the ER stress-related pathways modulate transcriptional programs and function to restore homeostasis, but the ultimate cellular outcome depends on the severity and duration of stress [4,5].

Lipopolysaccharide (LPS) injection induced ER stress in the livers of obese mice, which in turn resulted in liver injury via activation of the NLRP3 inflammasome [4], a multimolecular complex involved in the production of the caspase-1/-11-mediated proinflammatory cytokine interleukin (IL)-1β [6,7]. In the central nervous system, chronic activation of the UPR has been implicated in a diverse array of neurodegenerative diseases, potentially through neuroinflammation and synaptic dysfunction [3]. Neuronal ER stress upregulated the level of protein tyrosine phosphatase 1B (PTP1B), and this elevated PTP1B activity-inhibited signaling for neuronal circuits [8]. PTP1B was also involved in the production of proinflammatory cytokines in LPS-stimulated microglia via mediation of the stress-associated transcription factor NF-κB signaling [8,9]. Previous studies have also reported that ER stress within microglia was associated with proinflammatory phenotypes [10,11]. However, the mechanistic details underlying the pathological implications of ER stress and/or ER stress-related pathways in microglia inflammatory responses have not been clarified.

Oxytocin (OT) is a nonapeptide synthesized in the hypothalamus that acts as a hormone in the periphery for uterine contractions during parturition and milk ejection reflex [12,13,14]. It also behaves as a neurotransmitter within the brain for modulating social behaviors such as parental nurturing, social cognition, and social bonding [12,13,14]. In addition, OT has been implicated in various inflammatory responses in which it plays proinflammatory or anti-inflammatory roles depending on the cell type. In human myometrial and amnion cells, OT stimulation led to the production of proinflammatory cytokines through activation of the NF-κB signaling pathway [15,16]. In contrast, OT suppressed the production of LPS-induced proinflammatory mediators in mouse microglia via involvement with mitogen-activated protein kinases (MAPKs) but not the NF-κB signaling pathway [14,17]. Other studies have reported potential protective roles of OT for developing human enterocytes by modulating ER stress-related pathways in response to LPS stimulation [14,18]. However, the effects of OT on ER stress as well as on ER stress-related pathways in activated microglia remain to be elucidated.

In the present study, we investigated the effects of OT on the inflammatory response of LPS-stimulated microglia, focusing on the potential impact of OT on ER stress and stress-related pathways. The results here provide the first evidence of a novel anti-inflammatory axis mediated by OT within LPS-stimulated microglia.

## 2. Materials and Methods

### 2.1. Cell Culture and Treatments

The mouse microglial cell line MG6 (RIKEN Cell Bank, Tsukuba, Japan) was cultured in Dulbecco’s Modified Eagle’s Medium with high glucose (Sigma-Aldrich, Tokyo, Japan) containing 10% fetal bovine serum, 10 µg/mL insulin, and 100 µM β-mercaptoethanol [19,20,21]. The effects of OT (Abcam, Cambridge, UK) on inflammatory responses were investigated as follows: cells maintained at 37 °C were pretreated for 30 min with 1 µM OT (diluted in sterilized water) or a vehicle control, followed by stimulation with 100 ng/mL lipopolysaccharide (LPS) (Sigma-Aldrich, Tokyo, Japan) or LPS vehicle for 24 h in the continued presence of OT or the OT vehicle. To inhibit OT receptor (OTR) signaling, cells were pre-incubated for 30 min with the OTR antagonist (OTRA) L-371,257 (1 µM; Tocris Bioscience, Bristol, UK) [22], before adding OT and LPS. To inhibit PTP1B or ATF4 signaling, cells were transfected for 24 h with 15 pmol of PTP1B siRNA (sc-36329; Santa Cruz Biotechnology, Santa Cruz, CA, USA), ATF4 siRNA (sc-35113; Santa Cruz Biotechnology), or control scramble siRNA (sc-37007; Santa Cruz Biotechnology) using a Lipofectamine RNAiMAX (Life Technologies, Carlsbad, CA, USA) before treatment with OT and LPS. After washing, cells were harvested and subjected to further analyses.

### 2.2. Total RNA Extraction and Quantitative RT-PCR

We extracted total cellular RNA using the RNeasy Mini Kit (QIAGEN, Germantown, MD, USA), and synthesized first-strand cDNA using a High-Capacity RNA-to-cDNA Kit (Applied Biosystems, Foster City, CA, USA), according to the manufacturer’s instructions. To measure expression levels of the genes of interest, we performed quantitative RT-PCR using Power SYBR Green PCR Master Mix (Applied Biosystems) and the StepOnePlus Real-Time PCR System (Applied Biosystems, Foster City, CA, USA). The relative expression of each gene was determined by the 2^−ΔΔCt^ method. We used the expression levels of glyceraldehyde-3-phosphate dehydrogenase (GAPDH), 18S rRNA (18S), or acidic ribosomal phosphoprotein P0 (36B4) as internal controls as indicated. The following primer sequences were used: mouse tumor necrosis factor-alpha (TNF-α) forward primer (5′-GCCTCTTCTCATTCCTGCTTG-3′), reverse primer (5′-CTGATGAGAGGGAGGCCATT-3′) [23]; mouse IL-6 forward primer (5′-AGACAAAGCCAGAGTCCTTCA-3′), reverse primer (5′-GGTCCTTAGCCACTCCTTCTG-3′) [19]; mouse IL-1β forward primer (5′-CACAGCAGCACATCAACAAG-3′), reverse primer (5′-GTGCTCATGTCCTCATCCTG-3′) [24]; mouse GADD34 forward primer (5′-TTACCAGAGACAGGGGTAGGT-3′), reverse primer (5′-GAGGGACGCCCACAACTTC-3′) [24]; mouse CHOP forward primer (5′-CCACCACACCTGAAAGCAGAA-3′), reverse primer (5′-AGGTGAAAGGCAGGGACTCA-3′) [24]; mouse GAPDH forward primer (5′-TCCACTCACGGCAAATTCAACG-3′), reverse primer (5′-TAGACTCCACGACATACTCAGC-3′) [24]; mouse 18S forward primer (5′-AGGCCCAGAGCAAGAGAGGTA-3′), reverse primer (5′-GGGGTGTTGAAGGTCTCAAACA-3′) [25]; and mouse 36B4 forward primer (5′-TCCTTCTTCCAGGCTTTGGG-3′), and reverse primer (5′-GACACCCTCCAGAAAGCGAG-3′) [26] (Sigma-Aldrich, Tokyo, Japan).

### 2.3. Cytokine Measurements

Microglia were seeded in 24-well plates at a density of 1 × 10^5^ cells/mL (an equal number of cells in each well for normalization), followed by incubation with the vehicle, LPS, OT, and/or L-371,257, as indicated in the figures. Levels of TNF-α, IL-6, and IL-1β in the culture supernatant were measured using the Mouse TNF-α ELISA Kit (Proteintech, Rosemont, IL, USA), Mouse IL6 ELISA Kit (Proteintech, Rosemont, IL, USA), and Mouse IL1-β ELISA Kit (Proteintech, Rosemont, IL, USA), respectively, according to the manufacturer’s instructions.

### 2.4. Western Blot Analyses

Whole cell lysates were prepared with a lysis buffer containing 0.06 M Tri-HCl (pH 6.8), 2% sodium dodecyl sulfate (SDS), 10% glycerol, and 2.5% β-mercaptoethanol, supplemented with Halt™ Protease and Phosphatase Inhibitor Cocktail (Thermo Scientific, Tokyo, Japan). Total cellular protein (10 µg per each sample) was resolved by SDS-polyacrylamide gel electrophoresis, followed by transfer to polyvinylidene difluoride membranes. After treatment with a blocking solution (Nacalai Tesque, Kyoto, Japan), the membranes were incubated with one of the following rabbit primary antibodies: anti-phospho-NF-κB p65 (Ser536) (1:1000 dilution), anti-NF-κB p65 (1:1000), anti-phospho-p38 MAPK (Thr180/Tyr182) (1:1000), anti-p38 MAPK (1:1000), anti-phospho-PERK (Thr980) (1:1000), anti-PERK (1:1000), anti-phospho-eIF-2α (Ser51) (1:1000), anti-eIF-2α (1:1000), anti-ATF4 (1:1000), anti-ATF6 (1:1000), anti-GCN2 (1:1000), anti-β-actin (1:1000) (all from Cell Signaling Technology, Beverly, MA, USA), anti-phospho-IRE1α (Ser724) (1:1000), anti-IRE1α (1:1000) (Novus Biologicals, Centennial, CO, USA), anti-XBP1 (1:1000) (GeneTex, Irvine, CA, USA), anti-Caspase 1/p20/p10 (1:1000), anti-PTP1B (1:4000), anti-PKR (1:2000), anti-HRI (1:1000) (Proteintech, Rosemont, IL, USA), anti-phospho-PKR (Thr451) (1:2000) (Merck Millipore, Temecula, CA, USA), anti-phospho-GCN2 (Thr899) (1:1000) (Bioss, Boston, MA, USA), or anti-Caspase-11 (1:1500) (BioLegend, San Diego, CA, USA). The immunoreactive bands were detected with an HRP-conjugated anti-rabbit IgG secondary antibody (GE Healthcare, Uppsala, Sweden) and the ECL Prime Western Blotting Detection System (GE Healthcare, Uppsala, Sweden). We acquired gel images using the ChemiDoc MP imaging system (Bio-Rad, Hercules, CA, USA), and quantified protein expression levels by measuring band intensities using ImageJ (NIH, Bethesda, MD, USA).

### 2.5. Statistical Analysis

All data are expressed as mean ± SEM. Means were compared between the groups by Student’s *t*-test or one-way ANOVA with Tukey’s post hoc tests for pair-wise comparisons using SPSS v.22.0 for Windows (IBM Japan, Ltd., Tokyo, Japan). *P* values <0.05 were considered statistically significant.

## 3. Results

### 3.1. OT Suppression of LPS-Induced Proinflammatory Cytokine Production in Microglia

**Figure 1:**
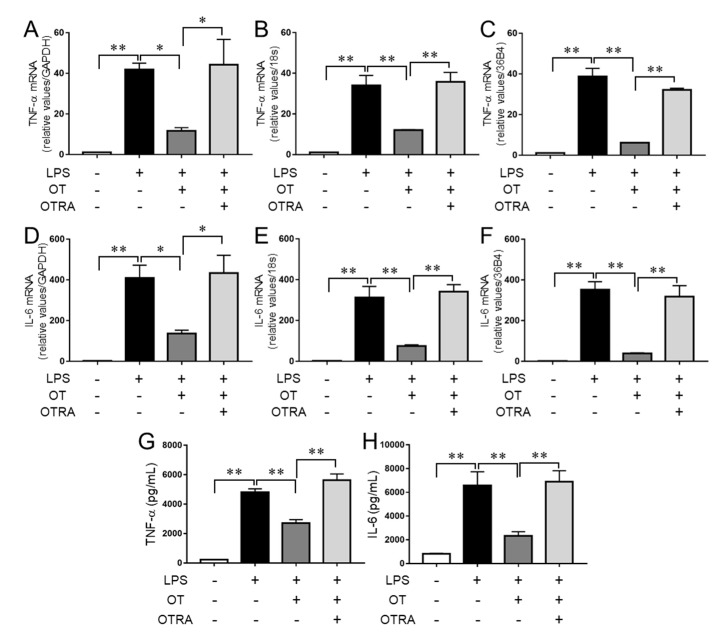
Oxytocin (OT) suppresses the production of proinflammatory cytokines in lipopolysaccharide (LPS)-stimulated microglia. MG6 microglial cells were pretreated with vehicle control, 1 µM OT, and/or 1 µM OT receptor antagonist (OTRA) for 30 min, followed by stimulation with LPS (100 ng/mL) for 24 h, as indicated. The mRNA expression levels of TNF-α (**A**–**C**) and IL-6 (**D**–**F**) were analyzed by quantitative RT-PCR and normalized to that of GAPDH (**A**,**D**), 18S (**B**,**E**), or 36B4 (**C**,**F**). Expression levels are displayed relative to vehicle-treated controls (1.0). The amounts of TNF-α (**G**) and IL-6 (**H**) in the culture supernatant were quantified using ELISA. Data are the mean ± SEM (*n* = 3 independent experiments). * *P* < 0.05; ** *P* < 0.01.

We previously demonstrated that the mouse microglial cell line MG6 exhibited inflammatory responses to LPS stimulation, which were suppressed by ω-3 polyunsaturated fatty acid (PUFA) treatment [19]. We further reported that another microglial cell line, namely BV-2, also displayed inflammatory responses to LPS stimulation, and ω-3 PUFAs inhibited the production of proinflammatory cytokines in LPS-stimulated BV-2 cells in a manner similar to that observed in MG6 cells [19]. Here we used MG6 microglia to investigate the effects of OT in response to LPS stimulation. As shown in Figure 1A, LPS stimulation resulted in significant elevation of gene expression levels of the proinflammatory cytokine TNF-α compared with controls and OT treatment significantly suppressed its expression, in line with a previous report [17]. We further found that the OT receptor antagonist (OTRA) L-371,257 [22] significantly reversed the suppressive effects of OT (Figure 1A). Similar results were obtained when 18S or 36B4 was used as an internal control (Figure 1B,C). Furthermore, the expression profiles of IL-6 were similar to those of TNF-α irrespective of the internal control used (Figure 1D–F). We therefore used GAPDH expression as an internal control in the following experiments to examine the expression levels of the genes of interest. We further confirmed that the protein production profiles of TNF-α and IL-6 were similar to those of the genes (Figure 1G,H). Additionally, we observed no significant effect of OT treatment alone or OTRA treatment alone on the levels of mRNA and protein of both TNF-α and IL-6 (Supplementary Figure S1). These results therefore indicated that OT treatment exhibits suppressive effects on proinflammatory cytokine production in LPS-stimulated MG6 microglia.

### 3.2. Effects of OT on the Activities of NF-κB and p38 MAPK in LPS-Stimulated Microglia

**Figure 2:**
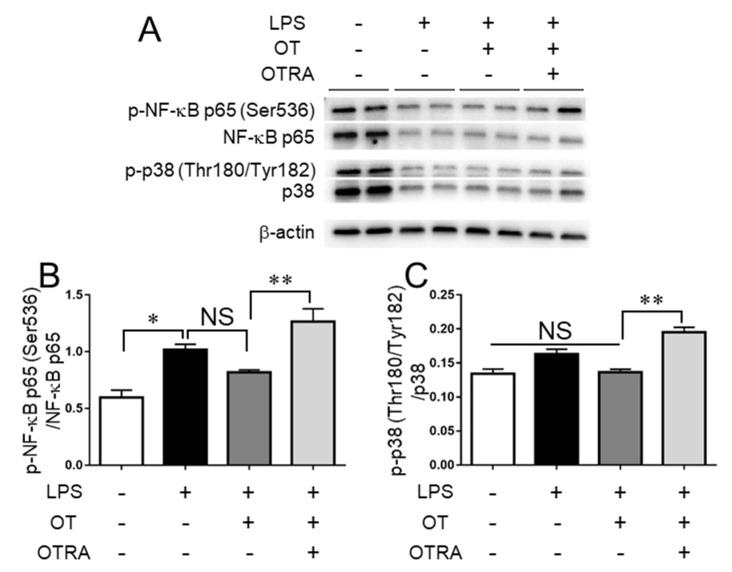
Oxytocin (OT) exhibits no remarkable effects on the phosphorylation levels of NF-κB subunit p65 at Ser536 and p38 MAPK at Thr180/Tyr182 in lipopolysaccharide (LPS)-stimulated microglia. MG6 microglia were treated for 24 h with the indicated reagent combinations. The amounts of phosphorylated NF-κB (p-NF-κB) p65 (Ser536), NF-κB, phosphorylated p38 (p-p38) (Thr180/Tyr182), p38, and β-actin were analyzed by western blot (**A**) and densitometry (**B**), the amount of p-NF-κB p65 relative to total NF-κB p65; (**C**), the amount of p-p38 relative to total p38. Images are representative of three independent experiments. Data are expressed as the mean ± SEM (*n* = 3 independent experiments). * *P* < 0.05; ** *P* < 0.01. NS, not significant. OTRA, OT receptor antagonist.

We next examined the effects of OT on the activation of NF-κB in LPS-stimulated MG6 microglia. LPS stimulation significantly increased phosphorylation levels of the NF-κB subunit p65 at Ser536, which enhances its transcription activity [27], but OT treatment exhibited no significant effect on phosphorylation levels (Figure 2A,B), in line with a previous report [17]. Conversely, LPS stimulation as well as OT treatment exerted no significant effect on the phosphorylation levels of p38 MAPK at Thr180/Tyr182 (Figure 2A,C), which is typically involved in its activity to produce inflammatory mediators [28] in our experimental conditions. After 24 h of incubation in the absence of LPS, NF-κB and p38 levels were sustained, and there was no significant effect of OT or OTRA treatment alone on the phosphorylation levels of these signaling molecules (Supplementary Figure S2). These results suggested that LPS downregulated the protein levels of NF-κB and p38 independent of OT and OTRA, and that OT suppressed the production of proinflammatory cytokines by inhibiting or alleviating other proinflammatory signaling in LPS-stimulated MG6 microglia.

### 3.3. Effects of OT on ER Stress and ER Stress-Related Pathways in LPS-Stimulated Microglia

A previous study has reported that LPS stimulation resulted in ER stress, which in turn induced inflammatory responses in mouse hepatocytes [4]. In addition, free cholesterol-induced ER-stressed mouse macrophages [29] as well as ER stress inducer-stimulated rat microglia [10] exhibited proinflammatory phenotypes. We therefore examined the effects of LPS stimulation on ER stress markers and whether OT treatment influenced ER stress and ER stress-related pathways in MG6 microglia.

**Figure 3:**
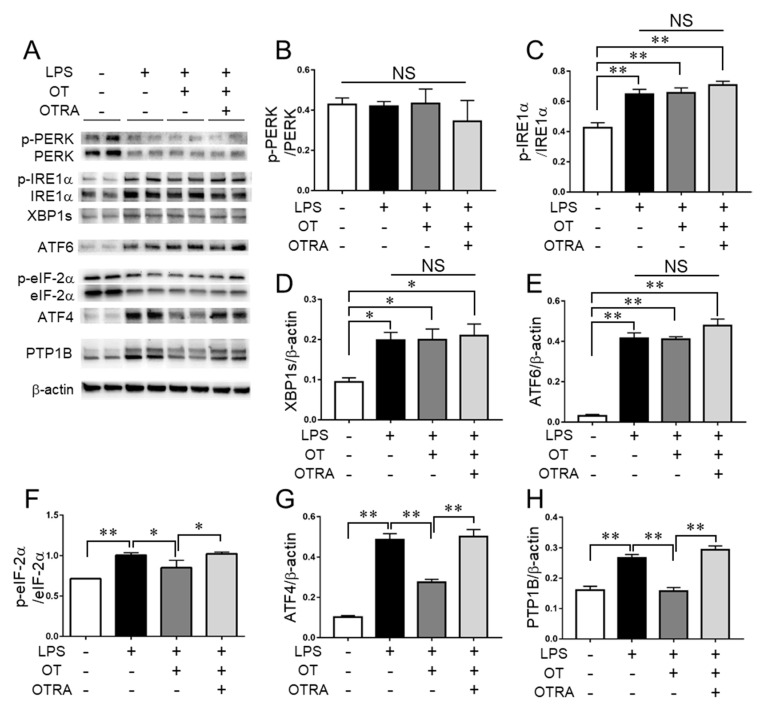
Oxytocin (OT) exerts no significant effects on ER stress sensors, but significantly reduces activation of a branch of ER stress-related pathways in lipopolysaccharide (LPS)-stimulated microglia. MG6 microglia were treated for 24 h with the described reagent combinations. The proteins of interest were analyzed by western blot (**A**) and densitometry (**B**–**H**). The images are representative of three independent experiments. Data are expressed as the mean ± SEM (*n* = 3 independent experiments). * *P* < 0.05; ** *P* < 0.01. NS, not significant. OTRA, OT receptor antagonist.

Although a previous study showed that LPS exerted a minimal effect on the levels of the ER stress sensor PERK [18], LPS downregulated the total amounts of PERK, whereas there were no significant differences in the phosphorylation levels of PERK between the groups (Figure 3A,B). Conversely, LPS led to significantly elevated phosphorylation levels of another ER stress sensor IRE1α compared with controls, but OT exerted no significant effect on IRE1α phosphorylation (Figure 3A,C). Similar results were obtained for the levels of XBP1s, a spliced form of XBP1 produced by activated IRE1α [5] (Figure 3A,D), as well as for another ER stress sensor ATF6 (Figure 3A,E). Thus, these results suggested that LPS elevated ER stress, but OT exhibited no remarkable effect on the ER stress levels in LPS-stimulated MG6 microglia.

We next examined the effects of LPS and OT on the ER stress-related pathways in MG6 microglia. LPS significantly elevated eukaryotic initiation factor-2α (eIF-2α) phosphorylation levels compared with controls (Figure 3A,F), which is an activated PERK-targeted attenuator of general translation in response to ER stress [5]. In addition, OT significantly suppressed the LPS-induced phosphorylation of eIF-2α, and OTRA significantly reversed its suppressive effects (Figure 3A,F), albeit no significant changes were observed for the phosphorylation levels of PERK (Figure 3A,B). We therefore further examined the effects of LPS and OT on the other three kinases that phosphorylate eIF-2α as follows: protein kinase double-stranded RNA-dependent (PKR), general control non-derepressible-2 (GCN2), and heme-regulated inhibitor (HRI) [30]. PKR, GCN2, and HRI respond to double-stranded RNA, amino acid deprivation, and heme deprivation, respectively [30]. Neither LPS nor OT affected the phosphorylation levels of either PKR or GCN2, and LPS elevated the total amounts of PKR and HRI (Supplementary Figure S3). Meanwhile, OT had no significant effect on the HRI levels (Supplementary Figure S3). We confirmed that OT or OTRA treatment alone resulted in no significant changes in the levels of these factors (Supplementary Figures S4A–F and S5). These results suggested that the additional kinase(s) employed for eIF-2α phosphorylation in response to LPS are inhibited by OTR signaling in LPS-stimulated MG6 microglia.

**Figure 4:**
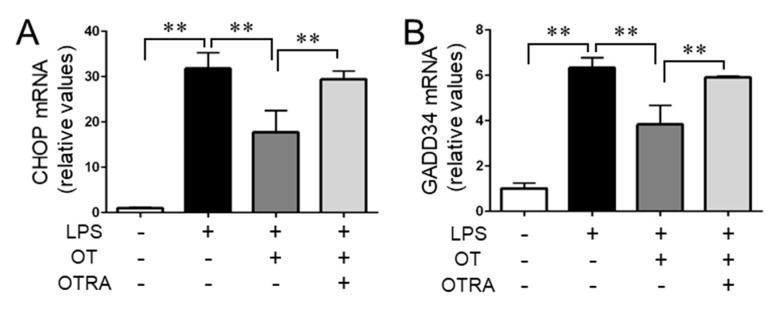
Oxytocin (OT) suppresses the expression of the ATF4 transcriptional targets CHOP and GADD34 in lipopolysaccharide (LPS)-stimulated microglia. MG6 microglia were treated for 24 h with the indicated reagent combinations. The mRNA levels of CHOP (**A**) and GADD34 (**B**) were analyzed via quantitative RT-PCR and normalized to that of GAPDH. Fold changes are displayed relative to those of vehicle-treated controls (1.0). Data are expressed as the mean ± SEM (*n* = 3 independent experiments). ** *P* < 0.01. OTRA, OT receptor antagonist.

In conjunction with the phosphorylation levels of eIF-2α, we found similar profiles in ATF4 levels (Figure 3A,G). ATF4 is a transcription factor whose translation is facilitated by p-eIF-2α, which enhances the transcription of CHOP and GADD34 [5,31]. We found that the gene expression profiles of both CHOP and GADD34 corresponded with ATF4 levels (Figure 3A,G and Figure 4A,B). Additionally, neither OT nor OTRA treatment alone had any significant effect on the levels of ATF4, CHOP, and GADD34 (Supplementary Figures S4G and S6). These results indicated that a branch of the ER stress-related pathways, i.e., the p-eIF-2α-ATF4 pathway, is activated by LPS and that OTR signaling suppresses its activation in LPS-stimulated MG6 microglia.

Previous studies have suggested that ER stress upregulates PTP1B, which in turn mediates cytotoxic signaling in neurons [8]. In microglia, PTP1B was elevated by LPS and involved in NF-κB-mediated inflammatory responses [8,9]. We found that LPS led to a significant increase in the level of PTP1B compared to controls, and that OT significantly suppressed the LPS-induced increase of PTP1B in MG6 microglia (Figure 3H). In addition, the suppressive effects of OT were significantly reversed by OTRA (Figure 3H). There was no significant effect of OT or OTRA treatment alone on the PTP1B levels (Supplementary Figure S4H). These findings suggested that OTR signaling exhibits suppressive effects on PTP1B in LPS-stimulated MG6 microglia.

### 3.4. Suppressive Effects of OT on Inflammasome Activation in LPS-Stimulated Microglia

**Figure 5:**
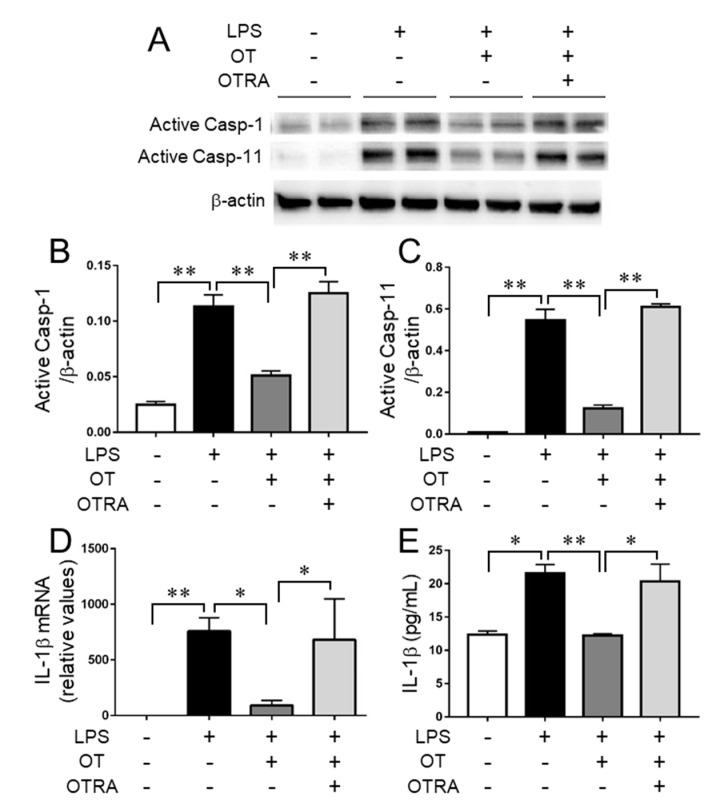
Oxytocin (OT) suppresses the activation of inflammasome in lipopolysaccharide (LPS)-stimulated microglia. MG6 microglia were treated for 24 h with the indicated reagent combinations. The amounts of active caspase-1 (Active Casp-1), active caspase-11 (Active Casp-11), and β-actin were analyzed by western blot (**A**) and densitometry (**B**, the amount of Active Casp-1 relative to β-actin; **C**, the amount of Active Casp-11 relative to β-actin). The IL-1β mRNA level (**D**) was determined by quantitative RT-PCR and normalized to that of GAPDH. Fold changes are shown relative to vehicle-treated controls (1.0). The amount of IL-1β (**E**) in the culture supernatant was measured using ELISA. The images are representative of three independent experiments. Data are expressed as the mean ± SEM (*n* = 3 independent experiments). * *P* < 0.05; ** *P* < 0.01. OTRA, OT receptor antagonist.

In mouse hepatocytes, LPS-induced ER stress increased the levels of ATF4, CHOP, and CHOP’s transcriptional targets caspase-1 and caspase-11 [4]. These in turn led to elevation of the formation of the active caspase-1/-11, producing IL-1β through NLRP3 inflammasome activation [4]. Furthermore, as previously described, LPS stimulation resulted in the activation of the ER stress-related p-eIF-2α–ATF4–CHOP pathway in MG6 microglia. We next investigated if the inflammasome was activated and whether OT was involved in attenuating its potential activation in LPS-stimulated MG6 microglia. LPS significantly increased the formation of both active caspase-1 and caspase-11 compared to controls, and OT treatment significantly suppressed these formations (Figure 5A–C). In addition, OTRA reversed the suppressive effects of OT (Figure 5A–C). In line with the activation statuses of caspase-1 and caspase-11, similar results were obtained for the gene expression levels as well as secreted protein levels of IL-1β (Figure 5D,E). We further confirmed the absence of a significant effect of OT or OTRA treatment alone on the levels of caspase-1, caspase-11, and IL-1β (Supplementary Figure S7). These results suggest the inflammasome activation and suppressive effects of OTR signaling on its activation in LPS-stimulated MG6 microglia.

### 3.5. Effects of OT on Proinflammatory Cytokine Production in PTP1B- or ATF4-Knockdown, LPS-Stimulated Microglia

**Figure 6:**
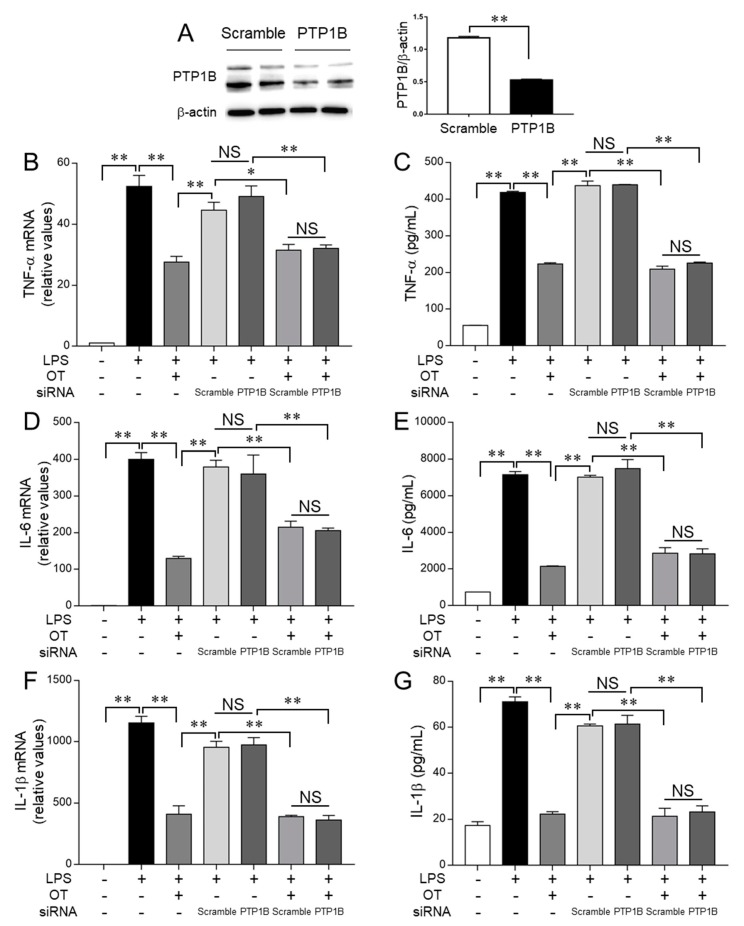
Oxytocin (OT) exerts no significant effects on PTP1B-related pathways in suppressing the production of proinflammatory cytokines in lipopolysaccharide (LPS)-stimulated microglia. MG6 microglia were treated for 24 h with the described reagent combinations after 24 h of transfection with control scramble siRNA or PTP1B siRNA. The amounts of PTP1B and β-actin 24 h after siRNA transfection (**A**) were analyzed via western blot (left panel) and densitometry (right panel). The mRNA expression levels of TNF-α (**B**), IL-6 (**D**), and IL-1β (**F**) were analyzed using quantitative RT-PCR and normalized to that of GAPDH. The expression levels are displayed relative to those of vehicle-treated controls (1.0). The amounts of TNF-α (**C**), IL-6 (**E**), and IL-1β (**G**) in the culture supernatant were quantified using ELISA. Data are expressed as the mean ± SEM (*n* = 3 independent experiments). * *P* < 0.05; ** *P* < 0.01. NS, not significant.

**Figure 7:**
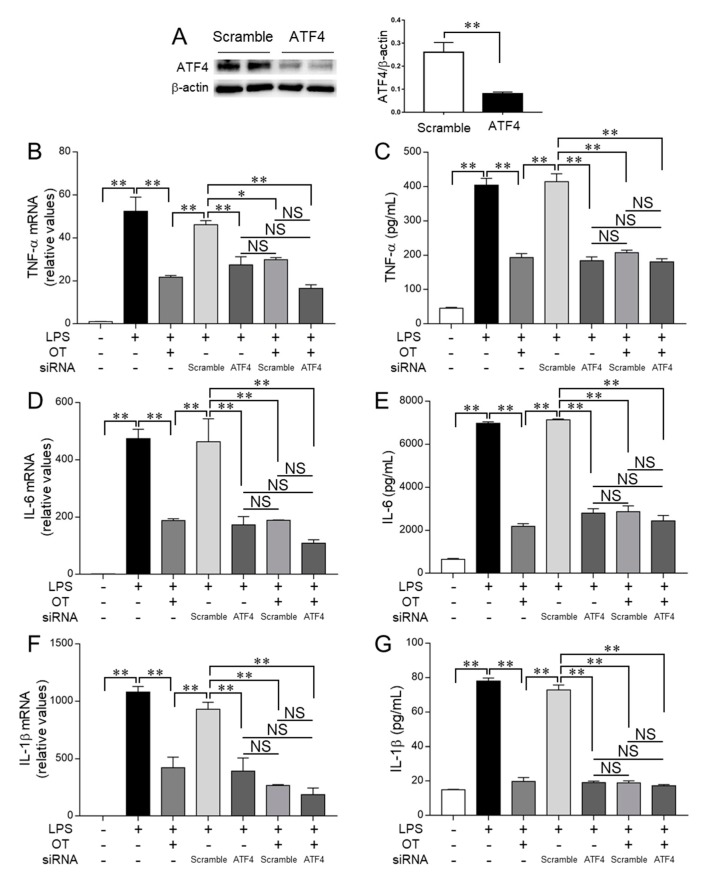
Oxytocin (OT) targets ATF4-related pathways to inhibit the production of proinflammatory cytokines in lipopolysaccharide (LPS)-stimulated microglia. MG6 microglia were transfected for 24 h with control scramble siRNA or ATF4 siRNA, followed by treatment for 24 h with the indicated reagent combinations. The amounts of ATF4 and β-actin 24 h after siRNA transfection (**A**) were analyzed via western blot (left panel) and densitometry (right panel). The mRNA expression levels of TNF-α (**B**), IL-6 (**D**), and IL-1β (**F**) were analyzed using quantitative RT-PCR and normalized to that of GAPDH. Fold changes are shown relative to those of vehicle-treated controls (1.0). The amounts of TNF-α (**C**), IL-6 (**E**), and IL-1β (**G**) in the culture supernatant were measured using ELISA. Data are expressed as the mean ± SEM (*n* = 3 independent experiments). * *P* < 0.05; ** *P* < 0.01. NS, not significant.

To validate the aforementioned findings, we next investigated the effects of OT on LPS-stimulated MG6 microglia, in which PTP1B or ATF4 was silenced using specific siRNA (Figure 6 and Figure 7).

If PTP1B is necessary for the production of TNF-α and/or IL-6 in LPS-stimulated MG6 microglia, then transfection with siRNA targeting PTP1B should attenuate the levels of these cytokines compared with the effects of transfection with control siRNA; however, PTP1B silencing had no effects on the levels of both TNF-α (Figure 6A–C) and IL-6 (Figure 6A,D,E) or on the levels of IL-1β (Figure 6A,F,G) in LPS-stimulated MG6 microglia, and OT also had no significant effect. Accordingly, these results suggest that PTP1B has a minor or no role in the production of these proinflammatory cytokines in LPS-stimulated MG6 microglia despite the findings that OT significantly suppressed PTP1B levels in these cells (Figure 3H).

Figure 7 shows the effects of OT on proinflammatory response to LPS in ATF4-knockdown MG6 microglia. Intriguingly, ATF4 silencing significantly reduced both the gene and protein levels of TNF-α compared with the effects of control siRNA transfection (Figure 7A–C), thereby suggesting a novel role of the p-eIF-2α–ATF4 pathway in the LPS-induced production of TNF-α. Furthermore, OT had no significant additive suppressive effect on the TNF-α levels in ATF4-knockdown cells (Figure 7B,C), suggesting that OT targets the p-eIF-2α–ATF4 pathway to reduce the production of TNF-α in LPS-stimulated MG6 microglia. In addition, we found that the effects of OT treatment and ATF4 silencing on IL-6 levels were similar to those on TNF-α levels (Figure 7D,E), thereby suggesting novel roles of the p-eIF-2α–ATF4 pathway in IL-6 production, which is also a novel target of OT for suppressing LPS-induced IL-6 production in MG6 microglia.

Regarding the effects of OT treatment and ATF4 silencing on the levels of IL-1β, we found that ATF4 silencing significantly reduced the gene and protein levels of IL-1β compared with the effects of control siRNA transfection, and OT treatment had no significant additive suppressive effect on IL-1β levels (Figure 7A,F,G). These results therefore suggest that OT targets the p-eIF-2α–ATF4 pathway to reduce IL-1β production in LPS-stimulated MG6 microglia.

## 4. Discussion

To the best of our knowledge, this is the first study to demonstrate that the p-eIF-2α-ATF4 pathway is involved in TNF-α and IL-6 production and that the p-eIF-2α-ATF4-CHOP pathway induces an inflammatory response through inflammasome activation in LPS-stimulated MG6 microglia. Furthermore, our results demonstrated that OT exhibited suppressive effects on the related inflammatory pathway. These findings therefore implicate the OT–p-eIF-2α–ATF4 axis as a novel pathway in the regulation of inflammatory responses in activated microglia.

**Figure 8:**
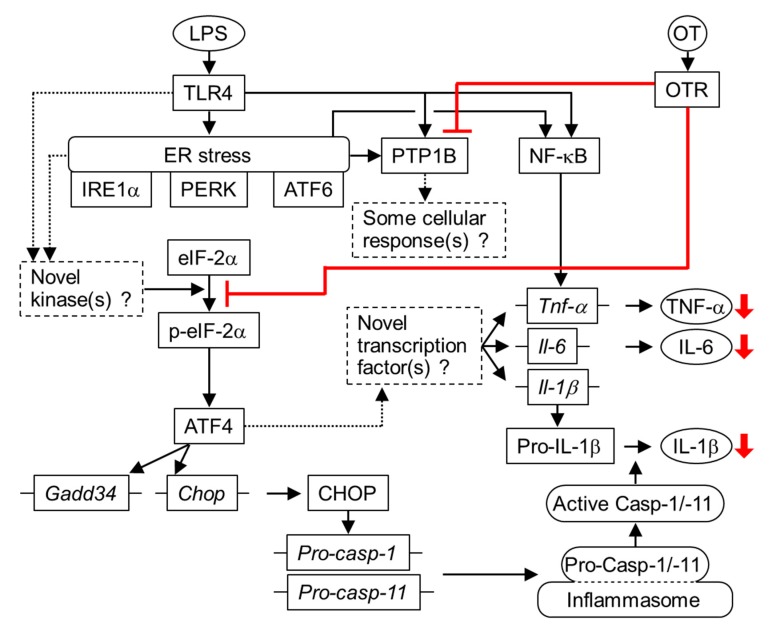
Potential mechanisms underlying the anti-inflammatory effects of oxytocin (OT) on lipopolysaccharide (LPS)-stimulated microglia. LPS binds its receptors, such as Toll-like receptor 4 (TLR4), increases ER stress, and stimulates inflammatory pathways for production of TNF-α, IL-6, and IL-1β in ER stress-dependent and -independent manners. In addition to NF-κB signaling, a branch of the ER stress-related pathways, namely the p-eIF-2α–ATF4 pathway, is also activated, stimulating unidentified transcriptional programs for the production of TNF-α, IL-6, and IL-1β. The p-eIF-2α–ATF4 pathway also upregulates CHOP levels, leading to the production of IL-1β mediated by inflammasome activation. OT binds to the OT receptor (OTR) and reduces PTP1B levels, which could contribute to modulating some cellular responses (red lines). Furthermore, OTR signaling inhibits the p-eIF-2α–ATF4 pathway, thereby suppressing the production of TNF-α, IL-6, and IL-1β (red lines).

Although previous studies have reported the proinflammatory roles of NF-κB and MAPKs, such as p38, within inflammatory responses in LPS-stimulated microglia [9,17,19], the mechanistic details underlying the implications of ER stress and/or ER stress-related pathways in microglial proinflammatory phenotypes remain unclear. In this respect, our study provided the first evidence that the p-eIF-2α–ATF4 ER stress-related pathway plays a novel role in producing proinflammatory cytokines through at least the two following distinct pathways: stimulation of the pathway for TNF-α and IL-6 production and activation of inflammasomes for IL-1β production. Our study further demonstrated that OT targets the p-eIF-2α-ATF4 pathway to suppress these proinflammatory axes. Of note, OT exerted no significant effect on the levels of the ER stress sensors PERK, IRE1α, and ATF6; hence, it is possible that OT has only minor roles in alleviating ER stress, per se, in LPS-stimulated microglia. Conversely, OT significantly reduced the phosphorylation levels of the PERK target eIF-2α and suppressed downstream signaling related to the production of TNF-α, IL-6, and IL-1β. Furthermore, OT had no significant effect on the activation of the other three known eIF-2α kinases, namely PKR, GCN2, and HRI. Accordingly, these findings suggested that OT inhibits additional kinase(s) targeting eIF-2α, which are activated by LPS and/or LPS-induced ER stress, leading to suppression of the proinflammatory axes in LPS-stimulated microglia (Figure 8).

In the present study, OT did not significantly reduce the activation of NF-κB and p38 but significantly suppressed the production of TNF-α and IL-6. These data therefore indicate that OT regulates potential novel transcriptional programs for the production of these proinflammatory cytokines, namely the p-eIF-2α–ATF4 pathway. In our experimental conditions, LPS stimulation for 24 h reduced the total amounts of NF-κB p65 and p38 compared with the effects of the vehicle control, whereas the amount of β-actin remained unchanged. Conversely, we previously observed that NF-κB p65 levels were comparable between 6 h of LPS stimulation and the vehicle control [19]. Accordingly, LPS stimulation for 24 h might trigger negative feedback mechanisms to attenuate some signaling molecules such as NF-κB, p38, and PERK in MG6 microglia. Future studies to address the temporal changes in the protein expression profiles of LPS-stimulated microglia would be helpful for better understanding the inflammatory responses of activated microglia.

A previous study reported that ER stress induced cytotoxic signaling via PTP1B activation in neurons [8]. Additionally, PTP1B is reportedly involved in NF-κB-mediated inflammatory responses in LPS-stimulated microglia [9]. However, our PTP1B silencing experiments suggested that PTP1B had a minor or no role in the production of TNF-α and IL-6 in LPS-stimulated microglia, whereas LPS upregulated PTP1B and OT suppressed its elevation. These findings suggest the previously unidentified pathophysiological significance of PTP1B and the potential roles of OT in modulating PTP1B-related cellular responses in activated microglia (Figure 8). Additional studies are required to address these issues.

Recent neurobiological studies have indicated the significance of OT in social and romantic relationships, which could be implicated in human health, including in mortality [13]. Physical contact is involved with OT release, and separation from a partner reduces OT synthesis [13]. Conversely, intranasal OT administration exhibited beneficial prosocial effects on patients with autism spectrum disorder [32,33], in which a microglial phenotypic dysfunction and neuroinflammation are implicated in the pathogenesis [34]. Accordingly, our findings may provide potential novel molecular rationales for OT’s clinical benefits; OT suppresses microglia-associated neuroinflammation, contributing to improvement and/or integrity of brain circuitries. This could also imply some beneficial effects of OT on dementia because it involves neuroinflammation and a decrease in social relationships is associated with an increased risk of this disease [35]. Future basic and clinical studies to elucidate in detail the actions of OT, OTRA, OTR agonists, and OT-related peptides are critical to address these issues.

In conclusion, the present study provided the first evidence that the p-eIF-2α–ATF4 pathway related to ER stress induces an inflammatory response in LPS-stimulated microglia. We further demonstrated the novel anti-inflammatory axis of OT in activated microglia. In this respect, our previous study illustrated that the ω-3 PUFAs eicosapentaenoic acid (EPA)/docosahexaenoic acid (DHA) exhibited suppressive effects on the inflammatory responses of activated microglia through the SIRT1-mediated inhibition of NF-κB signaling [19]. Furthermore, our most recent study using a mouse model of cerebral amyloid angiopathy, a cause of dementia, revealed that oral administration of the natural bioactive flavonoid taxifolin [36] suppressed the accumulation of activated microglia and improved inflammation in the brain [24]. Accordingly, concurrent treatment with OT and EPA/DHA or taxifolin may have synergistic beneficial effects on proinflammatory phenotypes of activated microglia. Future studies to elucidate the mechanistic details underlying the anti-inflammatory effects of OT as well as to identify novel key targets involved in OT signaling may reveal new avenues for improving microglial dysfunction, contributing to prevention and treatment of neuroinflammation-related diseases.

## Supplementary Materials

The following are available online at https://www.mdpi.com/2073-4409/8/6/527/s1.
