# Supplementary material for: Oxytocin Suppresses Inflammatory Responses Induced by Lipopolysaccharide through Inhibition of the eIF-2α–ATF4 Pathway in Mouse Microglia
Source: Cells. 2019 May 31;8(6):527. doi: 10.3390/cells8060527 (PMC6627458; doi:10.3390/cells8060527)
Supplement: Supplementary file 1 [file cells-08-00527-s001.pdf]

Cells

Supplementary Materials for

Oxytocin Suppresses Inflammatory Responses Induced by Lipopolysaccharide through Inhibition of the eIF-2α–ATF4 Pathway in Mouse Microglia

Takayuki Inoue, Hajime Yamakage, Masashi Tanaka, Toru Kusakabe, Akira Shimatsu and Noriko Satoh-Asahara

Corresponding author:
Masashi Tanaka
Email: masashi.7.tanaka@gmail.com

This PDF file includes Figs. S1 to S7.

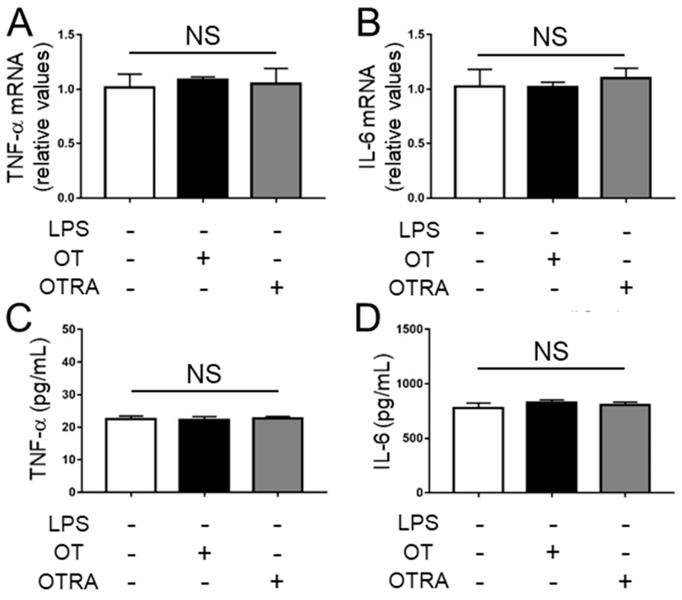

Figure S1. Effects of oxytocin (OT) or OT receptor antagonist (OTRA) on the production of proinflammatory cytokines in microglia. MG6 microglial cells were treated with vehicle control, 1 μM OT, or 1 μM OTRA for 24 h. The mRNA expression levels of TNF-α (**A**) and IL-6 (**B**) were analyzed using quantitative RT-PCR and normalized to that of GAPDH. The expression levels are displayed relative to those of vehicle-treated controls (1.0). The amounts of TNF-α (**C**) and IL-6 (**D**) in the culture supernatant were quantified using ELISA. Data are expressed as the mean ± SEM (*n* = 3 independent experiments). NS, not significant.

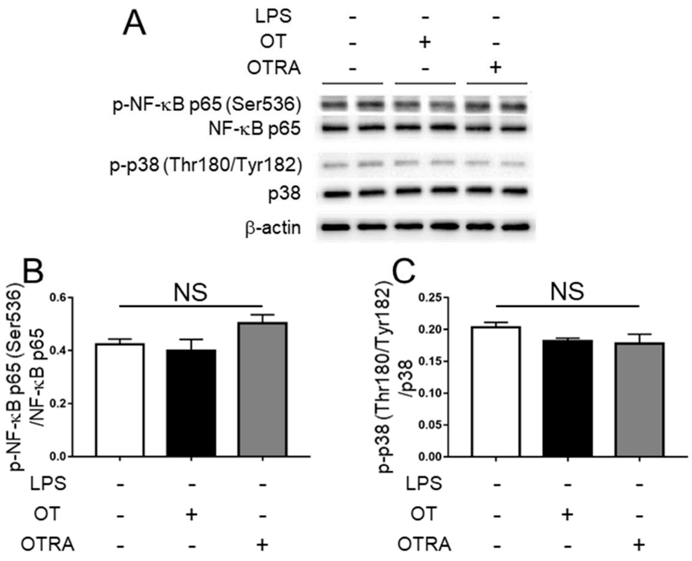

Figure S2. Effects of oxytocin (OT) or OT receptor antagonist (OTRA) on the phosphorylation of the NF-κB subunit p65 at Ser536 and p38 MAPK at Thr180/Tyr182 in microglia. MG6 microglia were treated for 24 h with OT or OTRA. The amounts of phosphorylated NF-κB (p-NF-κB) p65 (Ser536), NF-κB, phosphorylated p38 (p-p38) (Thr180/Tyr182), p38, and β-actin were analyzed via western blot (**A**) and densitometry (**B**, amount of p-NF-κB p65 relative to total NF-κB p65; **C**, amount of p-p38 relative to total p38). Images are representative of three independent experiments. Data are expressed as the mean ± SEM (*n* = 3 independent experiments). NS, not significant.

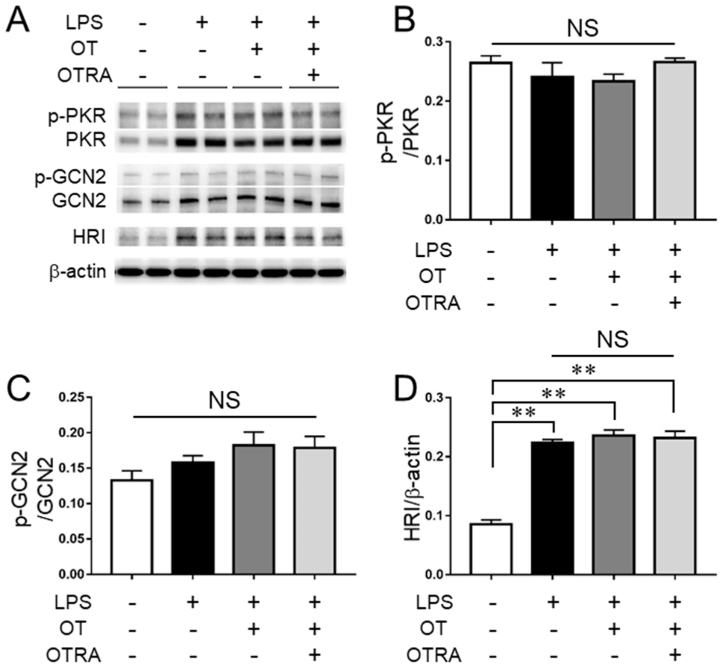

Figure S3. Effects of oxytocin (OT) on three kinases targeting eIF-2α in lipopolysaccharide (LPS)-stimulated microglia. MG6 microglia were treated for 24 h with the described reagent combinations. The proteins of interest were analyzed via western blot (**A**) and densitometry (**B**–**D**). The images are representative of three independent experiments. Data are expressed as the mean ± SEM (n = 3 independent experiments). ** $P < 0.01$. NS, not significant. OTRA, OT receptor antagonist.

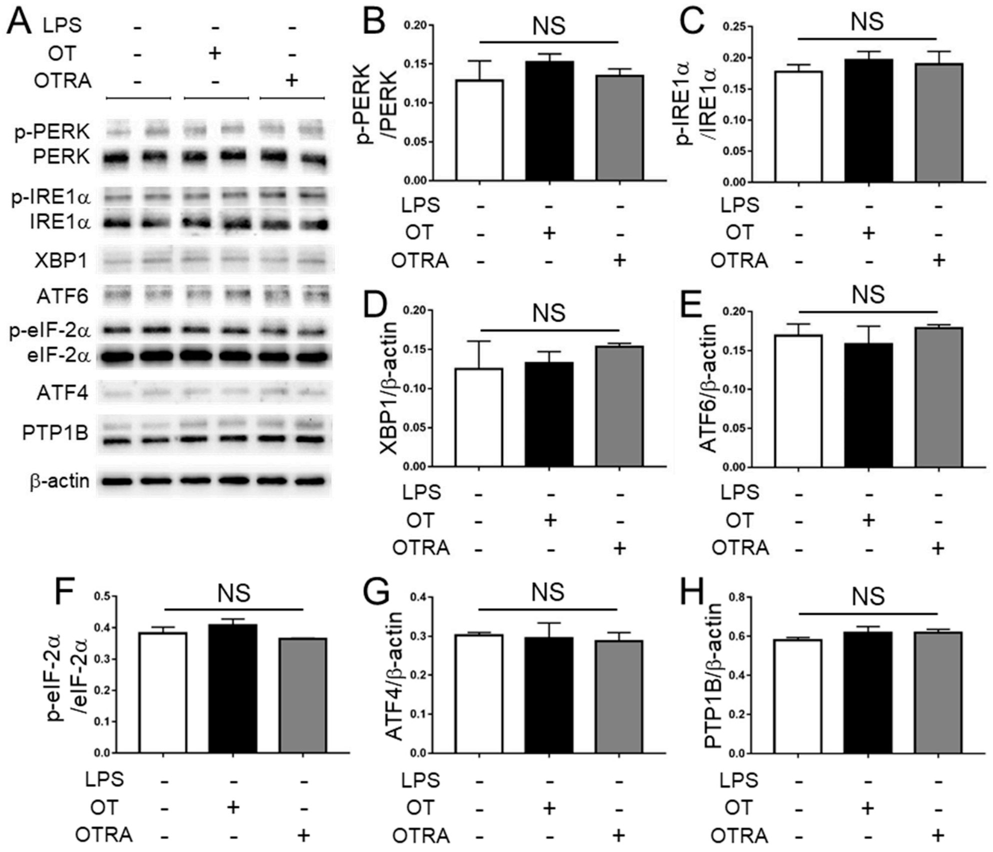

Figure S4. Effects of oxytocin (OT) or OT receptor antagonist (OTRA) on ER stress sensors and a branch of ER stress-related pathways in microglia. MG6 microglia were treated for 24 h with OT or OTRA. The proteins of interest were analyzed via western blot (**A**) and densitometry (**B**–**H**). The images are representative of three independent experiments. Data are expressed as the mean ± SEM (n = 3 independent experiments). NS, not significant.

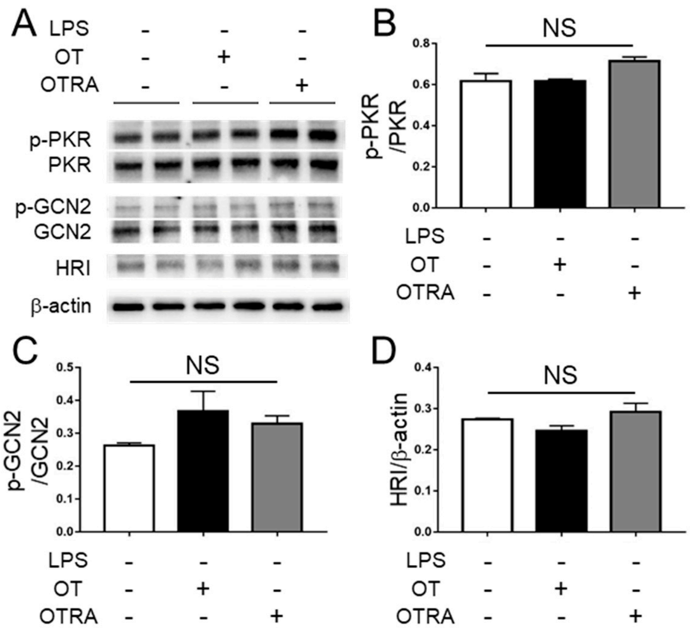

Figure S5. Effects of oxytocin (OT) or OT receptor antagonist (OTRA) on three kinases targeting eIF-2α in microglia. MG6 microglia were treated for 24 h with OT or OTRA. The proteins of interest were analyzed via western blot (**A**) and densitometry (**B**–**D**). The images are representative of three independent experiments. Data are expressed as the mean ± SEM (n = 3 independent experiments). NS, not significant.

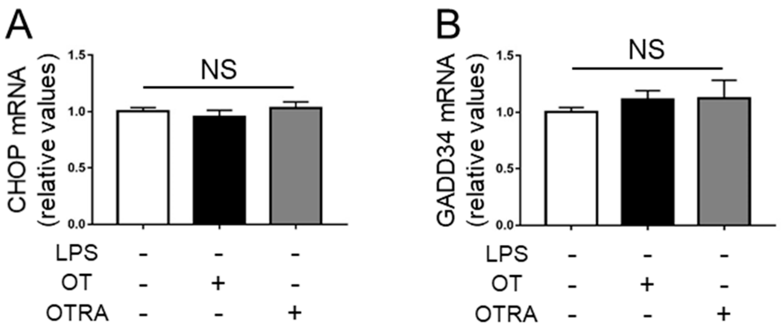

Figure S6. Effects of oxytocin (OT) or OT receptor antagonist (OTRA) on the expression levels of the ATF4 transcriptional targets CHOP and GADD34 in microglia. MG6 microglial cells were treated with vehicle control, 1 μM OT, or 1 μM OTRA for 24 h. The mRNA levels of CHOP (**A**) and GADD34 (**B**) were analyzed via quantitative RT-PCR and normalized to that of GAPDH. The expression levels are displayed relative to those of vehicle-treated controls (1.0). Data are expressed as the mean ± SEM (*n* = 3 independent experiments). NS, not significant.

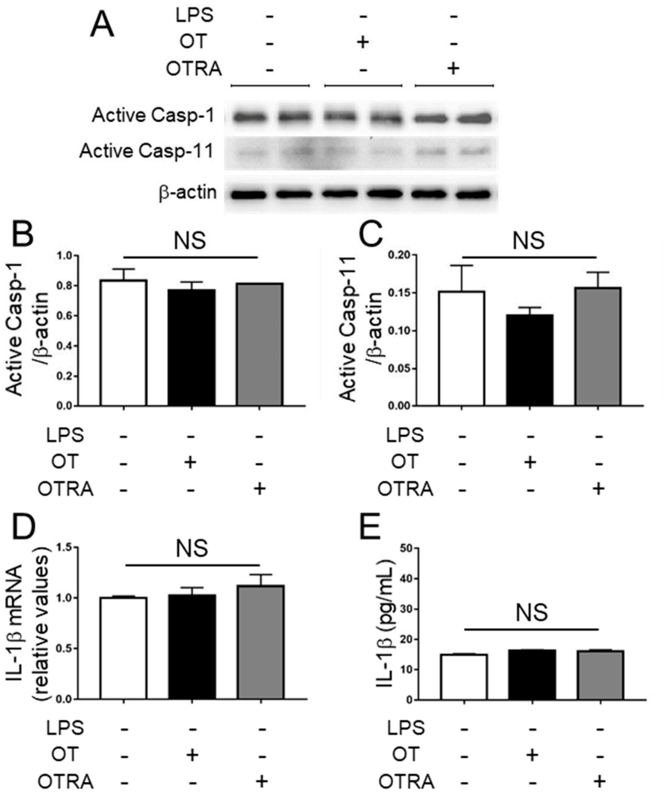

Figure S7. Effects of oxytocin (OT) or OT receptor antagonist (OTRA) on the activation of inflammasomes in microglia. MG6 microglia were treated for 24 h with OT or OTRA. The amounts of active caspase-1 (Active Casp-1), active caspase-11 (Active Casp-11), and β-actin were analyzed via western blot (**A**) and densitometry (**B**, amount of active Casp-1 relative to β-actin; **C**, amount of active Casp-11 relative to β-actin). The IL-1β mRNA level (**D**) was measured via quantitative RT-PCR and normalized to that of GAPDH. Fold changes are shown relative to those of vehicle-treated controls (1.0). The amount of IL-1β (**E**) in the culture supernatant was quantified using ELISA. The images are representative of three independent experiments. Data are expressed as the mean ± SEM (n = 3 independent experiments). NS, not significant.
